# Sjögren’s syndrome in children: about 15 cases in Guinea Conakry

**DOI:** 10.1186/s40001-021-00534-6

**Published:** 2021-07-02

**Authors:** Kaba Condé, Carlos Othon Guelngar, Mamadou Ciré Barry, Hugues Ghislain Atakla, Awada Mohamed, Fodé Abass Cissé

**Affiliations:** 1 Rheumatology Department, Ignace Deen University Hospital Center, Conakry, Guinea; 2 Neurology Department, Ignace Deen National Hospital, Conakry, Guinea; 3 Pediatric Department, Ignace Deen University Hospital Center, Conakry, Guinea; 4 Neurology Department, University Hospital Center Hubert Koutoukou MAGA, Cotonou, Benin

**Keywords:** Sjögren’s syndrome, Children, Conakry, Guinea

## Abstract

**Objectives:**

Sjögren’s syndrome is rare in children and most often secondary. It frequently affects girls and is characterized by dry eye syndrome, mouth and sometimes systemic involvement. Its diagnosis is difficult to establish in children. We report a series of 15 cases of Sjögren’s syndrome in order to clarify the peculiarities of this condition in children.

**Patients and methods:**

This retrospective study was carried out over a 2-year period focused on children under 16 years of age who had been followed for Sjögren’s syndrome in the rheumatology and pediatric departments. Patient data were collected and then analyzed by STATA/SE version 11.2 software. Anonymity and respect for ethical rules were the norm. There was no connection between the patients and the researchers.

**Description of cases:**

The mean age of the patients was 11 years with extremes of 5–15 years. History reveals that a dry mouth was found in more than half of the cases, or in 10 (66.7%) patients. Clinical examination found oral ulceration and periodontitis in equal proportions, 6 (40%). The immunological workup and the biopsy of the accessory salivary glands served as diagnostic evidence in the 15 patients according to the US–European criteria of 2002.

**Conclusion:**

Sjögren’s syndrome is a rare entity in pediatrics. Its diagnosis is difficult to establish in pediatrics and its severity is linked to the occurrence of late visceral and lymphomatous sicca syndrome. Rapid diagnosis and initiation of a synthetic antimalarial (hydroxychloroquine) increases the hope of a cure.

## Introduction

Sjögren’s syndrome is a rare autoimmune disease, most often secondary in children associated with other autoimmune diseases [1, 2]. It affects girls much more than boys [3] and is characterized by dry eye and mouth syndrome and in almost a third of patients the pathology is more systemic and can affect various organs [4]. Diagnosis is difficult in children given their inability to list their symptoms accurately and the difficulty in obtaining reliable data on the personal and family history of children. Diagnosis is based on a combination of clinical biological and immunological signs and biopsy of the accessory salivary glands [5, 6, 7]. A rapid diagnosis is necessary in order to prevent early complications, especially functionality related to the sicca syndrome and late such as visceral and lymphomatous [8].

The clinical manifestations frequently encountered are linked to an autoimmune exocrinopathy with xerostomia; xerophthalmia; tooth decay; periodontitis; dysphagia; dysgeusia and keratoconjunctivitis [9].

Despite the many diagnostic criteria for Sjögren’s syndrome, controversy persists as to the choice of a precise diagnostic criterion, especially in children. By adapting the American–European consensus group (CETA) classification, Bartounkova et al. have proposed a new scheme for the diagnosis of Sjögren’s syndrome in children [10–12]. We report 15 cases of childhood Sjögren’s syndrome.

## Patients and methods

This was a retrospective study carried out over a period of 2 years from August 15 2018 to August 15 2020. We targeted the records of patients (246) followed in the rheumatology and pediatrics department of the national hospital Ignace Deen, Conakry, for an SS. The study focused exclusively on children under 16 who were followed for SS. The diagnosis of SS was made in accordance with the American–European consensus criteria and Vitali’s criteria for secondary Sjögren syndrome. The data analyzed were the socio-demographic characteristics (age, sex), clinical (dry mouth, eye, skin, bronchial syndrome, ENT; articular, peripheral and axial involvement. Clinical forms: primary and secondary SS (associated with another autoimmune disease). Paraclinical data: immunological (anti-nuclear antibodies, anti-SSA and SSB antibodies) Biopsy of accessory salivary glands according to the classification of Chisholm and Mason). Patient data were collected and then analyzed by STATA/SE version 11.2 software. All parents and, if possible, capable children (aged 16) signed consent forms before being included in the study. It was clearly explained that if patients did not wish to participate in the study, it would not affect the quality of their care.

## Case presentation

Of the 15 patients followed in this study, eight were male and seven were female with an M/F sex ratio of 1.14. The average age of the patients was 11 years with extremes of 5–15 years. The history reveals dry mouth, respectively, in 10 (66.7%); of patients with Dry Eye Syndrome dominated by a foreign body sensation in the eyes; 8 (53.3%); 6 (40%) (Table 1). Clinical examination found oral ulceration in 6 (40%); periodontitis in 6 (40%); parotidomegaly in 5 (33.3%) (Table 1). Eye redness and photosensitivity were observed in comparable proportions (66.7%) (Table 1). The biology showed a normal blood count, a non-specific biological inflammatory syndrome with an average sedimentation rate 55 mm/h (range 5 and 130), as long as the C-reactive protein was positive with an average 35 mg/l (6 and 78). The immunological workup performed systematically in all patients shows an anti-SSA antibody in 6 children (40%) and anti-SSB antibody in 8 children (53.3%) (Table 1). Six (6) children or 40% were diagnosed with a grade IV Chisholm and Mason classification (Table 2). In addition, four children presented with SS with a grade III Chisholm and Mason classification (Table 2). Only three children or 6.27% had an SS with a Chisholm and Mason classification of normal grade. The clinical forms were dominated by the primary Sjögren syndrome nine cases as long as the secondary SS was present in six cases (rheumatoid arthritis four cases, lupus two cases). After an ophthalmological examination, the patients were put on hydroxychloroquine 500 mg/24 h.

**Table 1:** Socio-demographic characteristics of patients, clinical manifestations and paraclinical data of sicca syndrome

Variables	Effective (%) *N* = 15
Socio-demographic characteristics	
Sex	
Male	8 (53.3%)
Female	7 (46.7%)
Average age (extremes)	11 years (5 et 16 years)
Clinical manifestations	
Dry mouth	10 (66.7%)
Mouth ulceration	6 (40%)
Foods sticky to the mouth	4 (26.7%)
Mouth pain	2 (13.3%)
Hypo-ageusia	3 (20%)
Periodontitis	6 (40%)
Parotidomegaly	5 (33.3%)
Sensation of a foreign body in the eyes	8 (53.3%)
Eye burn	6 (40%)
Eye redness	9 (60%)
Photosensitivity	10 (66.7%)
No tear	3 (20%)
Nasal dryness	4 (26.7%)
Schimer test	9 (60%)
Immunological assessment	
Anti-SSA antibodies	6 (40%)
Anti-SSB antibodies	8 (53.3%)

**Table 2:** Classification of Chisholm and Mason

Chisholm-Mason grade	Effective	Percentage
Normal	3	20
Grade I	2	13.3
Grade II	0	0
Grade III	4	26.7
Grade IV	6	40
Total	15	100

## Discussion

Sjögren’s syndrome is a disease in adults with a high frequency in the fourth and fifth decades. It is rare and most often secondary in children and adolescents [9]. We report 15 cases of SS in children with a predominance of the primary forms and also noted a predominance of men. However, we find in the literature a predominance of women and secondary forms [1]. This finding in our study could be explained by the size of our sample (smaller).

Diagnosis research is difficult in children because of the often-atypical clinical presentation. The clinical manifestations listed in this study are the same commonly cited in the literature [10, 11, 13]. The American–European consensus criteria and that of Vitali allowed us to establish the diagnosis in the patients studied in this work. Houghton et al. [9] found 72.5% of recurrent periodontitis, in fact 40% of our patients have already presented with periodontitis. The occurrence of periodontitis is a fundamental element which increases the sensitivity of the European diagnostic criterion of Vitali et al. [9, 14].

The immunological assessment often reports the presence of anti-nuclear antibodies. Anti-SSA antibodies were positive in 40% of the children and anti-SSB antibodies in 53.3%. Hamzaoui et al. [11], report that anti-SSA antibodies are the most frequent in 54–75% of cases. The Schirmer’s test was positive (15 mm of wetting after 5 min) in more than half of the patients (60%). Treatment is primarily symptomatic with prevention of dental caries and ulcerative keratitis. Although no systemic therapy has so far proven to be effective, the synthetic antimalarial (hydroxychloroquine) used in our patients has had satisfactory results. Corticosteroid therapy and treatment with immunosuppressants can be initiated in cases of multi-visceral involvement.

## Conclusion

Sjögren’s syndrome is a rare entity in pediatrics; we report 15 cases with predominance of men and secondary forms. Its diagnosis can be difficult in children because of their inability to accurately describe their symptoms. And its severity is linked to the occurrence of dry and late syndromes such as visceral and lymphomatosis. Prompt diagnosis and induction of effective treatment will prevent complications.
